# Aliphatic Aldehydes in the Earth’s Crust—Remains of Prebiotic Chemistry?

**DOI:** 10.3390/life12070925

**Published:** 2022-06-21

**Authors:** Yildiz Großmann, Ulrich Schreiber, Christian Mayer, Oliver J. Schmitz

**Affiliations:** 1 Institute of Applied Analytical Chemistry (AAC), University of Duisburg-Essen, 45141 Essen, Germany; yildiz.grossmann@uni-due.de; 2 Teaching and Research Centre for Separation (TRC), University of Duisburg-Essen, 45141 Essen, Germany; 3 Department of Geology, University of Duisburg-Essen, 45141 Essen, Germany; ulrich.schreiber@uni-due.de; 4 Institute of Physical Chemistry, Center for Nanointegration Duisburg-Essen (CENIDE), University of Duisburg-Essen, 45141 Essen, Germany; christian.mayer@uni-due.de

**Keywords:** origin of life, hydrothermal solutions, fluid inclusions, aliphatic aldehydes

## Abstract

The origin of life is a mystery that has not yet been solved in the natural sciences. Some promising interpretative approaches are related to hydrothermal activities. Hydrothermal environments contain all necessary elements for the development of precursor molecules. There are surfaces with possible catalytic activity, and wide ranges of pressure and temperature conditions. The chemical composition of hydrothermal fluids together with periodically fluctuating physical conditions should open up multiple pathways towards prebiotic molecules. In 2017, we detected potentially prebiotic organic substances, including a homologous series of aldehydes in Archean quartz crystals from Western Australia, more than 3 billion years old. In order to approach the question of whether the transformation of inorganic into organic substances is an ongoing process, we investigated a drill core from the geologically young Wehr caldera in Germany at a depth of 1000 m. Here, we show the existence of a similar homologous series of aldehydes (C_8_ to C_16_) in the fluid inclusions of the drill core calcites, a finding that supports the thesis that hydrothermal environments could possibly be the material source for the origin of life.

## 1. Introduction

Naturally occurring aliphatic aldehydes may play an important role as potentially prebiotic precursor molecules. With growing chain lengths, the so-called fatty aldehydes gain amphiphilic properties, and could serve as starting points for a large collection of membrane-forming lipids [1,2]. The reactivity of the aldehyde head group allows a wide variety of chemical modifications, including oxidation to fatty acids; reduction to alcohols; the formation of amines and amides; and, finally, ester or ether formation with glycerol [3]. Even though the corresponding amines and carboxylic acids have not been detected, there are well known and direct links to these compounds via reduction, oxidation, or reactions with ammonia. Therefore, it can be assumed that they have been present as well in the original hydrothermal environment.

They still play a role in the recent biosynthesis of sphingolipids, where a long-chain aldehyde is undergoing a condensation reaction with serine [4].

The biochemical formation of long aliphatic chains with a hydrophilic head group is quite complicated and involves a considerable amount of energy; therefore, it is not very likely to have occurred as an initial development. On the other hand, early metabolic processes required functional membranes at an early point of time. Therefore, during the early steps of life, they very likely depended on an external occurrence of such molecules. All theories on the origin of protocells which are based on initial membrane formation require an abiotic source of amphiphilic components [5,6,7].

Recently, aliphatic aldehydes have been discovered in carbon-rich chondritic meteorites [8]. Aponte et al. analyzed ten different carbonaceous chondrites and, using a novel analytical approach, detected and quantified 16 different aldehydes. Among them were butanal, pentanal, and hexanal in concentrations around 2–6 nmol/g. Most aldehydes in chondrites exhibit a clear enrichment of ^13^C isotopes compared to their biogenic counterparts, a finding which indicates their abiotic formation [8]. This result is highly interesting because it represents convincing proof for an abiotic (in this case, extraterrestrial) origin of such potentially amphiphilic compounds, which are possible starting points for membrane-forming lipids. The analytical method used to analyze the aldehydes was based on derivatization with (S,S)-(-)-1,4-dimethoxy-2,3-butanediol ((S,S)-DMB-diol). The analysis ended after 95 min, and the derivatized hexanal was detected at a retention time of approximately 93 min; therefore, any longer-chain aldehydes present were not observable.

Moreover, there are also clear indications for a terrestrial abiotic formation of aldehydes. A complete family of homologous aliphatic aldehydes of even and uneven chain lengths was detected inside Archean quartz crystals [9]. Inclusions in these Archean quartz crystals grown in a hydrothermal environment contained eleven different species, from heptanal up to heptadecanal. This terrestrial evidence is even more intriguing concerning early membrane formation because of the wide and continuous distribution of chain lengths and the fact that the chain functionalization occurs almost exclusively on the methyl end groups of the chain. Due to the selected analytical conditions, the analysis of short-chain aldehydes was not possible here. Results from stable isotope analysis of the methane content of the inclusions are in clear accordance with an abiotic formation of the detected hydrocarbons [9].

It is likely that analyses using both analytical methods would detect both short-chain and long-chain aldehydes in meteorites and quartz samples. This assumption is supported by the results of the recent paper by Mißbach et al. [10]. The authors used chromatographic methods to investigate the constituents of fluid inclusions in 3.5-billion-year-old barites, which—just like the quartz samples already studied in 2017 by Schreiber et al.—originated from Western Australia. It is particularly noteworthy that the authors were only able to detect short-chain aldehydes here, namely ethanal, propanal, pentanal, and heptanal, based on the analytical parameters.

Thus, it could be shown that a prebiotic presence of short- and long-chain aldehydes is not exclusively due to meteorite impacts on the early Earth, but also due to hydrothermal formations, probably based on Fischer–Tropsch-type reactions.

Altogether, the abiotic formation of aldehydes may be a common feature of planetary bodies in, at least, our solar system. At this point, one has to ask the question of if it is an ongoing process that occurs up to the present day. If it is, one should be able to find evidence for abiotically formed aliphatic aldehydes in recent hydrothermal environments. To minimize contamination by metabolites from microbial life in a corresponding search, sampling should occur outside of densely populated environments. On the other hand, it should focus on materials that are in close contact with hydrothermal fluids, and that could be able to collect possible organic products over an extended period of time.

For that purpose, a drilling project was started in the phonolite/trachyte complex of the Wehr caldera (Eifel mountains) southwest of Cologne, near the lake, “Laacher See” (50°25′35.139″ N, 7°13′10.4322″ E). In this volcanic environment, we expect a rich flow of hydrothermal fluids consisting of water and carbon dioxide as the bulk solvents. Under the conditions which occur deep in the Earth’s crust, we assume a large variety of organic compounds to form [9]. Among other reactions, Fischer–Tropsch-type chemistry should lead to aliphatic chains, which are expected to undergo partial oxidation on their methyl end groups, eventually leading to a homologous series of aldehydes [11,12]. At approximately 1 km of depth, carbon dioxide is expected to undergo a phase transition from the supercritical to the sub-critical gaseous phase. During that transition, the carbon dioxide essentially loses its capability to act as a hydrophobic solvent [13]. As a consequence, this leads to the precipitation of mostly hydrophobic products at this point, forming an accumulation zone of corresponding organic compounds in this depth range [14]. In the following, we want to report on analytical data obtained from fluid inclusions in the solid core material from 1 km depth.

## 2. Materials and Methods

### 2.1. The Drill Core Sample

**Figure 1:**
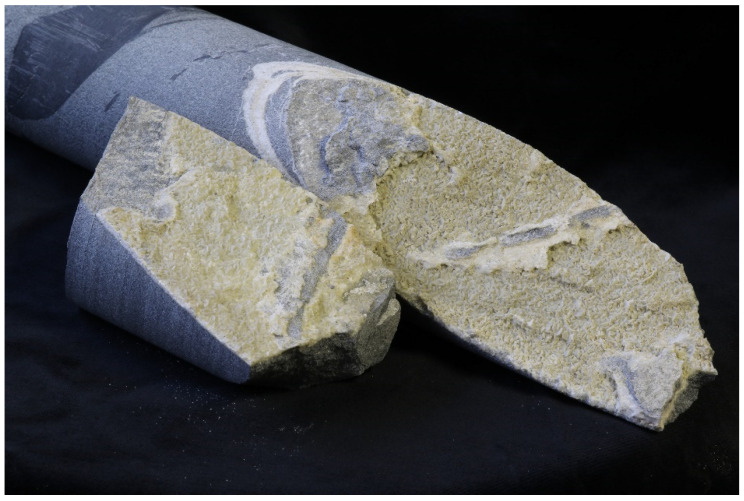
Light-colored calcite in the fragmented drill core.

A drill core from the Wehr caldera (N 50°43′, E 7°22′ East Eifel volcanic field, Germany) was taken out of the depth of 950 m to 968 m. The Wehr volcano erupted twice, and the most recent eruption took place 150,000 years ago [15]. This eruption formed a collapse structure in the Devonian basement [16], which was filled up with various layers of tephra and sediments. The hydrothermal processes are closely linked to volcanic activity, which is also evident in the Devonian basement. In particular, the built Quaternary hydrothermal calcite is of relevant importance here. There are fluid inclusions inside the hydrothermal calcites which are highly interesting for the possible occurrence of organic compounds. For this purpose, samples of hydrothermal calcite from a drilling core were taken. Hydrothermal fluids may have penetrated into cracks in the fine-grained Devonian rocks (clayish shale, silt stone, fine-grain sandstone), possibly leading to the crystallization of idiomorphic calcites (Figure 1). Further information about the geological framework is shown in Supplementary Information S1.

### 2.2. Sample Preparation

The calcite samples were taken mechanically by fragmentation of the drill core under protected conditions. After the cleaning procedure of the sample surface with hexane, the washed calcite sample was cooled with liquid nitrogen, mortared with a mortar and a pestle, and extracted three times with 10 mL hexane. The solution of the grinding step was collected in a Teflon tube. The mortared calcite (powder) was immediately scraped out of the mortar with the pestle and collected in the same Teflon tube. The resulting suspension was centrifuged with an Eppendorf Centrifuge 5804 R for 10 min at 3000 rpm and 14 °C. The supernatant was enriched with a Büchi Syncore apparatus for seven hours with 200 rpm at 40 °C to 1000 µL. Then, 500 µL of the 1000 µL sample were taken for the analysis of volatile organic compounds with higher vapour pressure (HVOCs). The residual 500 µL were enriched to dryness and resolved with 100 µL hexane for the analysis of volatile organic compounds with a lower vapour pressure (LVOCs). Finally, the samples were analyzed with a GC Q-TOF MS. Further information about the sample preparation is shown in Supplementary Information S2.

### 2.3. Determination of Organic Compounds by GC Q-TOF MS

GC Q-TOF MS analyses were performed on a gas chromatograph equipped with quadrupole time-of-flight gas chromatography (7890B GC/Q-TOF coupled to an accurate mass analyzer (MS 7250), both from Agilent (Santa Clara, CA, USA)). Ionization was performed by electron impact (70 eV). Data acquisition was performed and processed by MassHunter software, version B.08.00, from Agilent Technologies (Santa Clara, CA, USA), and NIST Library (Gaithersburg, MD, USA). Further information about the chromatographic and mass spectrometric setup is listed in Supplementary Information S3.

## 3. Results

**Figure 2:**
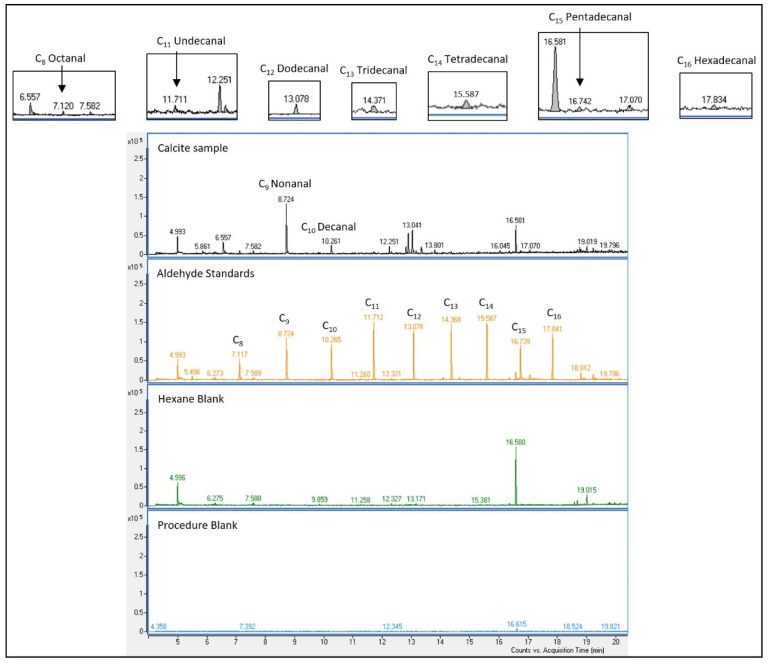
EIC of the [C_7_H_11_]^+^ aldehyde fragment with m/z 95.0864 of the calcite sample in comparison to the aldehyde standards, and hexane and procedure blank.

The focus of the analyses was on the detection of aldehydes in the calcite samples of the drill core. Figure 2 shows the result of the calcite sample in comparison to those of the aldehyde standards, and the hexane and procedure blanks.

The organic content in calcite samples shows, at least, minor concentrations of aldehydes in the fluid inclusions of the calcite in the core sample. All measurements are triple measurements, and the aldehydes were identified with standards. The quantification was made by external calibration (Supplementary Information S4 and S5). Table 1 shows the results of the measurements. Further information, such as mass spectra, are shown in the Supplementary Information S5.

By means of a six-point external calibration procedure, it was possible to determine the concentration of the detectable aldehydes in the calcite sample. Obviously, it has to be taken into account that this is not a representative sample, since the drill core cannot represent a homogeneous environment. One calcite sample was analyzed in three replicates, and the concentration of each aldehyde in the sample was calculated between approximately 10 and 600 µg/kg (details in Table 2). The concentrations of aldehydes in the calcite sample are in the same concentration range as those of the Archean quartz sample with 4–33 µg/kg [9], and of the aldehydes present in various meteorites [8].

## 4. Discussion

Durham et al. described a supercritical Fischer–Tropsch synthesis using a potassium-promoted iron-based catalyst and supercritical hexane, which allows the production of large amounts of long chain aldehydes with more than ten carbon units [17]. Whether this reaction is also possible in supercritical CO_2_ has not yet been clarified, but in our opinion, it is likely. Though alcohols and carboxylic acids were detected in the Fischer–Tropsch-type experiments in a hydrothermal system by McCollom et al. [18] with montmorillonite as a common mineral (sodium aluminum silicate), we have detected the intermediate oxidation state in the form of aldehydes (heptanal to heptadecanal) in fluid inclusions of Archean quartz samples [9] and calcites (octanal to hexadecanal) from a 1000 m deep drill core. These findings are supported by the work of Mißbach et al. [10], who detected short-chain aldehydes in fluid inclusions of 3.5-billion-year-old barites with a method which does not allow the analysis of long-chain aldehydes. In addition, aldehydes were also detected in various meteorites in a similar concentration range. Aponte et al. [8], who also used a method that does not allow the analysis of long-chain aldehydes, were able to detect short-chain aldehydes in various carbonaceous chondrites. However, these results indicate that the aldehydes found in meteorites could possibly be of extraterrestrial hydrothermal origin.

Just like in the Fischer–Tropsch-type experiments of McCollom et al., we detected a lack of an even/odd carbon number predominance, and a decreasing abundance of compounds with an increasing number of carbon atoms. The experiments conducted by McCollom et al. with and without montmorillonite, and in glass, as well as new steel vessels, led to the conclusion that montmorillonite does not act so much as a classical catalyst in the Fischer–Tropsch-type reaction. Instead, the reaction products were removed from the reaction mixture by adsorption to the montmorillonite, which drives the reaction to the product side according to the Le Châtelier principle [18]. This assumption is supported by a work from Iuga and Vivier-Bunge, who investigated the physisorption of small aliphatic aldehydes on a model silicate *Brønsted* site by using quantum chemical methods [19]. The *Brønsted* site was formed by a large silicate cluster model of 15 Si atoms formed by four six-membered rings. In the central siloxane bridge, one Si atom was replaced by a tetra-coordinated Al atom. The authors identified two different types of physisorption complexes for aldehydes. In both, there is a hydrogen bonding interaction between the hydrogen atom of the acid surface and the aldehydic carbonyl group. The aliphatic chain can be oriented perpendicular or parallel to the surface. In the first case, there is an additional van der Waals interaction between an adjacent siloxane bridge and the aldehydic hydrogen atom. In the second case, corresponding interactions occur between siloxane bridges and aliphatic hydrogen atoms of the aldehyde. The results presented in this work suggest that extended silica surfaces with *Brønsted* defects, such as pyrophyllite, can lead to a strong absorption of aldehydes [19]. In addition, Hakim et al. demonstrated that simple organic molecules, such as propanol or octanoic acid, form ordered adsorption layers on calcite, where the layer thickness is defined by their character. They postulated that organic compounds from the surrounding environment would adhere to the surface [20].

The formation of aldehydes is very interesting, since alkylamines can be formed very easily from aldehydes, e.g., via a reaction with ammonia in the presence of formic acid (similar to a Leuckart–Wallach reaction) or alkylcarboxylic acids via an acid-catalyzed reaction. Both ammonia and various acids occur in higher concentrations in hydrothermal fault zones [13].

Mayer et al. proposed a mechanism of periodic vesicle formation which is expected to occur in fault zones filled by water and CO_2_ [21]. At a depth of approximately 1 km, pressure and temperature conditions induce a local phase transition between supercritical CO_2_ (scCO_2_) and subcritical gaseous CO_2_ (gCO_2_). Under these conditions, the presence of amphiphilic compounds, such as alkyl amines or alkyl carboxylic acids, under these conditions inevitably leads to the transient formation of coated water droplets in the gas phase, and corresponding vesicular structures in the aqueous environment. The process of periodic formation and destruction of vesicles simultaneously provides a perfect environment for molecular evolution in small compartments, and for the emergence of protocells. The basic process of vesicle formation was experimentally reproduced using a mixture of octadecylamine and octadecanoic acid with a mass ratio of 1:1 in a water/CO_2_ system [22,23,24].

## 5. Conclusions

The investigations of fluid inclusions in calcite samples yield strikingly similar results as the analyses of Archean quartz samples from Western Australia, or recent analyses on chondritic meteorites: a homologous series of long-chain aldehydes. Returning to the question of whether the hydrothermal conversion of inorganic (CO, H_2_, H_2_O) to organic compounds, such as aldehydes, is an ongoing process that continues to occur today, we believe that our results demonstrate that this is, in fact, the case.

## Figures and Tables

**Table 1:** Identified aldehydes in fluid inclusions of calcite taken from Wehrer Kessel in Volcanic Eifel, Germany.

Aldehyde	Chemical Formula	Mass [g/mol]	Rt_sample_ [min]	RSD_Rt_ (*n* = 3) [%]	Rt_ref_ [min]	NIST [%]
Octanal	C_8_H_16_O	128.2144	7.12	0.02	7.12	90.8
Nonanal	C_9_H_18_O	142.2413	8.72	0.02	8.72	89.2
Decanal	C_10_H_20_O	156.2682	10.26	0.01	10.27	88.8
Undecanal	C_11_H_22_O	170.2951	11.71	0.02	11.71	90.1
Dodecanal	C_12_H_24_O	184.3220	13.08	0.01	13.08	81.7
Tridecanal	C_13_H_26_O	198.3449	14.37	0.01	14.37	91.2
Tetradecanal	C_14_H_28_O	212.3715	15.59	0.01	15.59	80.7
Pentadecanal	C_15_H_30_O	226.3981	16.74	0.07	16.74	81.7
Hexadecanal	C_16_H_32_O	240.4247	17.83	0.08	17.84	86.0

**Table 2:** Concentration of aldehydes in the fluid inclusions of the calcite sample (more details in Supplementary Table S3).

Aldehyde	Area	c [µg/kg]	RSD [%]
Octanal	15,220	109	12.0
Nonanal	203,139	582	2.1
Decanal	35,050	142	2.6
Undecanal	5851	23	11.2
Dodecanal	111,912	362	14.2
Tridecanal	6554	31	16.7
Tetradecanal	3420	18	1.9
Pentadecanal	6169	53	3.1
Hexadecanal	5813	36	13.3

## Data Availability

All presented data are available in the Applied Analytical Chemistry at the University of Duisburg-Essen, Germany.

## Supplementary Materials

The following supporting information can be downloaded at: https://www.mdpi.com/article/10.3390/life12070925/s1.
