# Supplementary material for: Aliphatic Aldehydes in the Earth’s Crust—Remains of Prebiotic Chemistry?
Source: Life (Basel). 2022 Jun 21;12(7):925. doi: 10.3390/life12070925 (PMC9319801; doi:10.3390/life12070925)
Supplement: Supplementary file 1 [file life-12-00925-s001.zip › life-1755745-supplementary.pdf]

Supplementary Information

Aliphatic aldehydes in the Earth's crust – remains of prebiotic chemistry?

Yildiz Großmann[1,2], Ulrich Schreiber[3], Christian Mayer[4], Oliver J. Schmitz[1,2,*]

[1]Institute of Applied Analytical Chemistry (AAC), University of Duisburg-Essen, 45141 Essen, Germany;

[2]Teaching and Research Centre for Separation (TRC), University of Duisburg-Essen, 45141 Essen, Germany;

[3]Department of Geology, University of Duisburg-Essen, 45141 Essen, Germany;

[4]Institute of Physical Chemistry, Center for Nanointegration Duisburg-Essen (CENIDE), University of Duisburg-Essen, 45141 Essen, Germany.

***Corresponding author:** Oliver J. Schmitz
Applied Analytical Chemistry, University of Duisburg-Essen
Universitaetsstrasse 5, 45141 Essen, Germany
Phone: +49 201 183-3950
Email: oliver.schmitz@uni-due.de

This PDF file includes:

Supplementary Text
Supplementary References
Supplementary Figures S1 to S4
Supplementary Tables S1 to S3

S1 Geological framework of the Wehr well

The nearly 1000 m deep well was drilled in 2019 in a mofette area of the Wehr caldera in the Quaternary volcanic field of the Osteifel, Germany, in 2019 by CARBO, Bad Hönningen (UTM coordinates: 32 U 373522 m E, 5587551 m N). The caldera is a volcano-tectonic collapse structure formed by two Plinian eruption cycles (Plinian eruptions within eruptive cycles of phonolitic and trachytic tephra) with the eruption of phonolitic and trachytic tephra 215 and 151 ka ago [1]. An 18 m long drill core with a diameter of 7 cm was recovered from a depth of between 950 and 968 m. It spans exactly the depth range where the boundary temperature of 31 °C and thus the transition from gCO_2 to $scCO_2$ lies. The core consists of steeply inclined, alternating layers of Devonian sandstones, siltstones, and clay stones of the Rhenish Massif. It has open fissures with idiomorphic grains of calcite. Small amounts of fluid from the deep fluid system are trapped inside the calcite crystals.

S2 Sample preparation

S2.1 Cleaning procedure of the working materials

Before using the ADA-1 Vapour cleaner (Figure S1) a self-cleaning procedure was performed for 24 hours. Before sample preparation, all materials (glass materials, mortar, pestle, and Teflon tubes) were carefully cleaned with nitric acid 65 % in an ADA-1 Vapour cleaner. All materials were then rinsed six times with suprapure water to pH = 7 and then dried in an oven at 100 °C for

24 to 48 hours to remove water. Before use, all materials were rinsed three times with hexane. Blanks were taken from each material and were analyzed by GC Q-TOF MS.

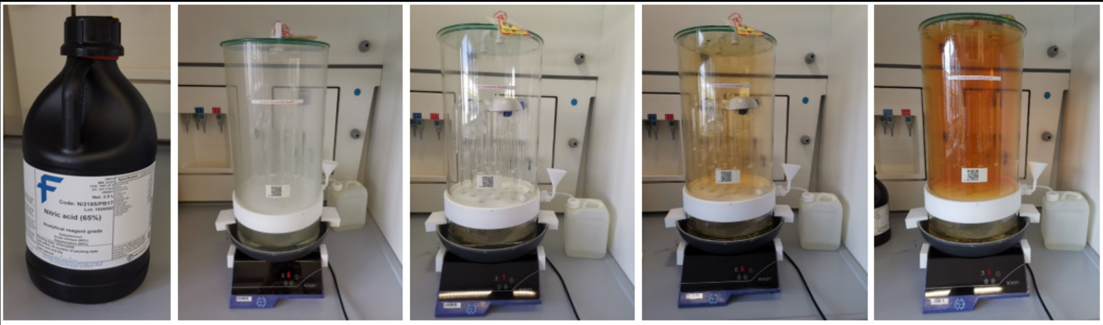

Figure S1. ADA-1 Vapour cleaner and the cleaning procedure of all materials.

S2.2 Procedure blank (sample-free)

A sample-free process was performed to exclude contamination. For the process blank, 10 mL hexane was added with a 10 mL glass pipette to a mortar (sample-free) three times and the sample grinding process was imitated for 5 min with a pestle. Liquid nitrogen was added in parallel. The solution of each grinding step was collected in a Teflon tube and centrifuged for 10 min at 3000 rpm at 14 °C. The supernatant was immediately placed in a Büchi Syncore system and concentrated to 1000 µL. 500 µL were taken and analyzed for the high volatile organic compounds (HVOCs). The remaining 500 µL were concentrated to dryness. Subsequently, the low volatile organic compounds (LVOCs) were resolved with 100 µL hexane and two 50 µL aliquots of each sample were prepared for analysis.

S2.3 Cleaning procedure of the sample surface

Figure S2 shows the cleaning and sample preparation procedure of the calcite surface to eliminate any contaminations from the outside.

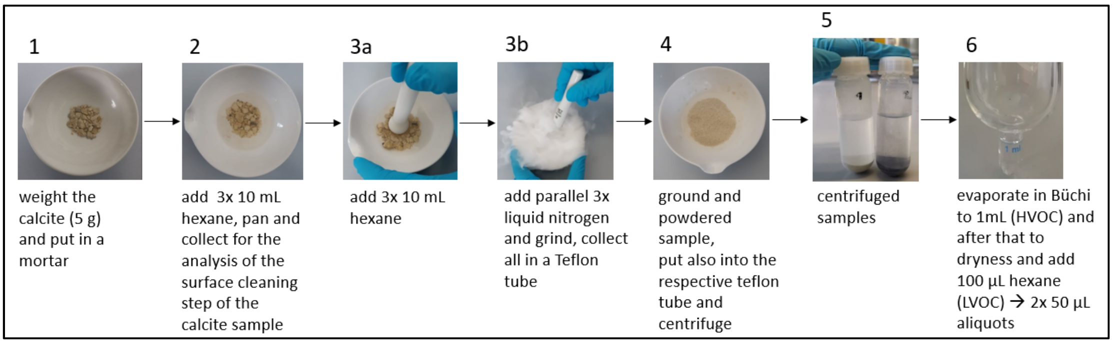

Figure S2. Procedure of sample preparation.

5 g calcite were washed three times with 10 mL hexane at room temperature for 5 min by swivelling. The calcite-hexane-washing-solution was used as a blank and analyzed with the GC Q-TOF MS system. After removing the hexane for the blank, 3 x 10 mL hexane were added to the washed calcite and the calcite sample was grinded under liquid nitrogen and finally, the powder, together with the hexane, were collected in a Teflon tube and centrifuged for 10 min at 3000 rpm at 14 °C. The supernatant was immediately placed in a Büchi-Syncore system and concentrated to 1000 µL. 500 µL were taken and analyzed for the high volatile organic compounds (HVOCs). The remaining 500 µL were concentrated to dryness. Subsequently, the low volatile organic compounds (LVOCs) were resolved with 100 µL hexane and two 50 µL aliquots of each sample were prepared for analysis.

S2.4 Standards mix preparation

3 mg of each aldehyde were weighed into a vial. To this, 5 mL of hexane was added. The vials were then placed in an ultrasonic bath for 20 min to allow the aldehydes to dissolve. Then everything was combined into a 100 mL flask and filled up to 100 mL with hexane. This leads to a concentration of 45.5 mg/kg. 110 µL of this solution was added to a 10 mL flask and filled up to 10 mL with hexane. That causes a concentration of 0.5 mg/kg for the working solution.

S2.5 Verification by standards

In Table S1 all aldehyde standards are listed. The aldehyde standards (C_8-C_{13}) are from Sigma Aldrich (St. Louis, MO, United States), aldehyde standards (C_{14}-C_{15}) are from abcr GmbH (Karlsruhe, Baden-Württemberg, Germany) and aldehyde standard C_{16} is from Tokyo Chemical Industry (TCI) (Tokio, Prefekture Tokio, Japan).

Table S1. Aldehyde standards

Aldehyde	Chemical Formula	Mass [g/mol]	$RT_{Calcite\ sample}$ [min]	$RT_{Standard}$ [min]
Octanal	$C_8H_{16}O$	128.2144	7.12	7.12
Nonanal	$C_9H_{18}O$	142.2413	8.72	8.72
Decanal	$C_{10}H_{20}O$	156.2682	10.26	10.27
Undecanal	$C_{11}H_{22}O$	170.2951	11.71	11.71
Dodecanal	$C_{12}H_{24}O$	184.3220	13.08	13.08
Tridecanal	$C_{13}H_{26}O$	198.3449	14.37	14.37
Tetradecanal	$C_{14}H_{28}O$	212.3715	15.59	15.59
Pentadecanal	$C_{15}H_{30}O$	226.3981	16.74	16.74
Hexadecanal	$C_{16}H_{32}O$	240.4247	17.83	17.84

S3 GC Q-TOF MS Method.

Table S2 shows the GC- and MS-parameters.

Table S2. GC Q-TOF MS Method for the analysis of the calcite sample

GC MS			
**GC 7890B**		**MS 7250**	
Temperature program rate:	40 °C (1 min), 10 °C/min to 300 °C (5 min)	EI:	70 eV
Carrier gas:	Helium 5.0	Ion source temp.:	200 °C
Flow:	1 mL/ min	ACQ Mode:	Fullscan
Mode:	splitless	Acquisition:	40-500 m/z
Injection volume:	1 µL	Acquisition Rate:	5 spectra/ s
Heater temp. (Injection):	280 °C	Acquisition Time:	200 ms/ spectra
Aux. temp. (Transfer Line):	310 °C	Solvent delay:	4 min
Column:	Rxi-5MS (30 m x 250 µm x 0.25 µm)	Mass error:	< 5 ppm
Run time:	32 min		

S4 Quantification with the external calibration

The quantification was done with a six-point external calibration procedure. The regression lines for all detected aldehydes are presented in the following nine plots (Figure S3).

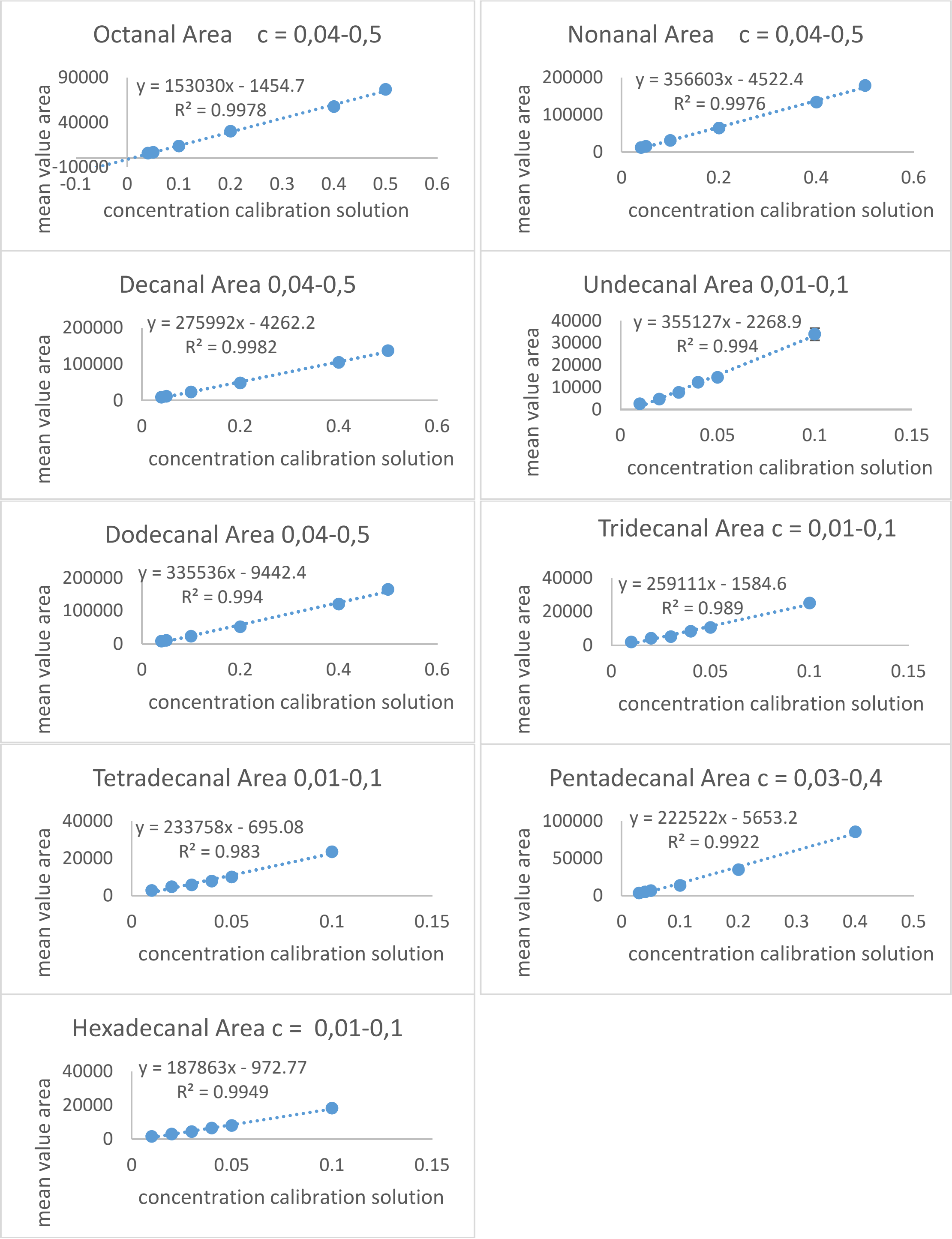

Figure S3. Six-point external calibration of the aldehydes.

S5 Results

S5.1 Aldehyde analysis in calcite samples

One calcite sample was analyzed with three replicates. At the same day, aldehyde standard solutions with concentrations between 0.01 – 0.5 mg/ kg were analyzed (n = 3). Based on these data, the concentration of each aldehyde in the calcite sample (Table S3) was calculated from the corresponding regression curve.

Table S3. Concentration of aldehydes in the fluid inclusions of the calcite sample

Replicate	Aldehyde	Area	c [mg/kg]	Mean value c [µg/kg]	RSD [%]
1	Octanal	14570.95	0.105	109	12.0
2		13615.31	0.098		
3		17472.47	0.124		
1	Nonanal	204132.56	0.585	582	2.1
2		198321.69	0.569		
3		206964.18	0.593		
1	Decanal	33859.31	0.138	142	2.6
2		35731.72	0.145		
3		35559.61	0.144		
1	Undecanal	5291.39	0.0213	23	11.2
2		5358.90	0.02148		
3		6903.05	0.02583		
1	Dodecanal	96989.45	0.31720	362	14.2
2		108061.67	0.35020		
3		130684.55	0.41762		
1	Tridecanal	5051.72	0.02561	31	16.7
2		6903.42	0.03276		
3		7707.44	0.03586		
1	Tetradecanal	3343.62	0.01712	18	1.9
2		3502.24	0.01780		
3		3412.87	0.01742		

1	Pentadecanal	6490.20	0.05457	53	3.1
2		6252.86	0.05351		
3		5764.02	0.05131		
1	Hexadecanal	5095.58	0.03230	36	13.3
2		6825.67	0.04151		
3		5518.58	0.03455		

S5.2 Mass spectra

As an example, Figure S4 shows the spectra comparison of nonanal in the sample and standard.

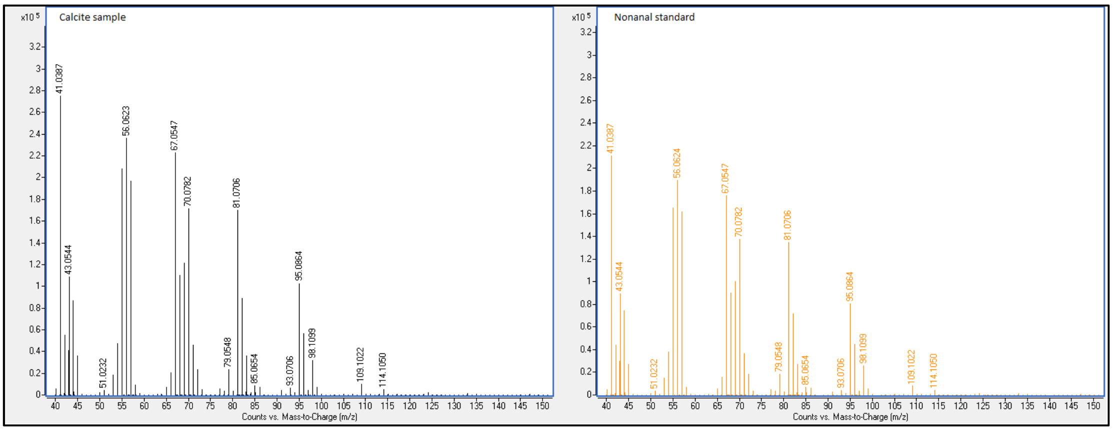

Figure S4. Comparison of MS spectra of nonanal in the calcite sample with the nonanal standard.

Supplementary References

[1] van den Bogaard, P.; Hall, C. H.; Schmincke, H.-U.; York, D. Precise single-grain 40Ar/39Ar dating of a cold to warm climate transition in Central Europe. *Nature* **1989,** *342*, 523–525.
